# Draft Genome Sequence of Methylocystis heyeri H2^T^, a Methanotroph with Habitat-Specific Adaptations, Isolated from a Peatland Ecosystem

**DOI:** 10.1128/MRA.00454-19

**Published:** 2019-07-18

**Authors:** Igor Y. Oshkin, Kirill K. Miroshnikov, Svetlana N. Dedysh

**Affiliations:** a Winogradsky Institute of Microbiology, Research Center of Biotechnology RAS, Moscow, Russia; University of Delaware

## Abstract

Methylocystis heyeri H2^T^ is an aerobic facultative methanotroph which was isolated from an acidic Sphagnum peat bog lake and is a common inhabitant of peatland ecosystems. This bacterium possesses two particulate methane monooxygenases with low and high affinity to methane and a number of genomic adaptations to acidic conditions.

## ANNOUNCEMENT

Methylocystis species are one of the numerically dominant and metabolically active methanotroph populations in northern wetlands. However, only two peat-inhabiting members of this genus, Methylocystis heyeri and Methylocystis bryophila, are available in cultures (1). Here, we report the draft genome sequence of Methylocystis heyeri H2^T^, a moderately acidophilic methanotroph of the class *Alphaproteobacteria* and family *Methylocystaceae*. It was isolated from the acidic (pH 4.3) Sphagnum peat bog lake Teufelssee in Germany (2). Cells of *M. heyeri* H2^T^ are Gram-negative, nonmotile, and encapsulated large rods or ovoids that multiply by binary fission. Strain H2^T^ utilizes methane and methanol but is also capable of slow growth on acetate, which supports the facultative nature of this methanotroph (3).

Strain H2^T^ was cultivated in a liquid mineral medium, M2, as described by Dedysh et al. (4). DNA was extracted from liquid culture using the AmpliSens DNA-sorb-AM kit (Russia). DNA was sheared to 20 kb utilizing needle shearing and used to generate large SMRTbell libraries. The libraries were further size selected utilizing BluePippin (Sage Scientific, Beverly, MA), with a cutoff size of 10 kb, and were sequenced using single-molecule real-time (SMRT) sequencing technology on a PacBio RS II system (Pacific Biosciences, Menlo Park, CA, USA). A total of 84,411 reads were obtained, with a mean read length of 3,850 bp. Error correction and trimming were performed using Canu 1.7 (5). *De novo* assembly was done using SPAdes 3.11.1 (6). Bioinformatic procedures were performed using the default parameters. The total length of the final assembly was 4,691,083 bp; it consisted of 12 genomic contigs with an *N*_50_ value of 3,287,266 bp. The estimated size of the M. heyeri H2^T^ genome is 4.7 Mb (coverage, 69×), with an average G+C content of 63.0%. In total, 4,247 predicted protein-coding genes were identified. The assembled contigs were annotated using the RAST server (7), BlastKOALA (8), and Prokka (9).

The genome of *M. heyeri* H2^T^ encodes two types of particulate methane monooxygenases, pMMO1 and pMMO2, and soluble MMO and contains two additional copies of the *pmoC* gene, encoding the γ-subunit of pMMO. The presence of high-affinity pMMO2 indicates the ability to grow at a low methane concentration (10). Growth on methanol is explained by the presence of the gene operons encoding MxaFI- and XoxF-type methanol dehydrogenases (11). Genes encoding pyrroloquinoline quinone cofactor biosynthesis proteins, tetrahydromethanopterin-linked and tetrahydrofolate-mediated pathways, and NAD-linked formate dehydrogenase were identified. A complete set of genes involved in the function of the serine cycle for formaldehyde assimilation was present. The complete ethylmalonyl-coenzyme A (ethylmalonyl-CoA) pathway and aldehyde and alcohol dehydrogenases detected in the genome provide the possibility for growth on acetate or ethanol. The presence of the genes encoding the molybdenum-iron (Mo) and vanadium-iron (V) types of nitrogenase provides the ability for nitrogen fixation. The V-nitrogenase is found only in a limited number of microorganisms, including another peat-inhabiting methanotroph (12). Acid resistance mechanisms identified in the genome include potassium uptake systems such as Trk- and Kef-type potassium transporters and a Kup-type low-affinity potassium transporter, as well as a Kdp-type high-affinity potassium transporter.

### Data availability.

This whole-genome shotgun project has been deposited in GenBank under the accession number SOPH00000000 (BioProject accession number PRJNA528078). The PacBio reads are available in the SRA under accession number SRP189702. The version described in this paper is the first version, SOPH01000000.
